# Absence of low back pain in patients followed weekly over one year with automated text messages

**DOI:** 10.1186/2045-709X-20-9

**Published:** 2012-03-29

**Authors:** Charlotte Leboeuf-Yde, Rikke K Jensen, Iben Axén

**Affiliations:** 1 Research Department, Spine Center of Southern Denmark, Hospital Lillebælt and Institute of Regional Health Services Research, University of Southern Denmark, Østre Hougvej 55, Middelfart, Denmark; 2 Karolinska Institutet, Intervention & Implementation, Research Institute of Environmental Medicine, Nobelsväg 13, Stockholm S- 171 77, Sweden

## Abstract

**Background:**

In order to define the onset of a new episode of low back pain (LBP), the definition of a "non-episode" must be clear. De Vet et al reviewed the scientific literature but found no evidence-based definitions of episodes or non-episodes of LBP. However, they suggested that pain-based episodes should be preceded and followed by a period of at least one month without LBP. As LBP is an episodic disease, it is not clear whether a sufficient number of patients with LBP will be LBP-free for at least one month ("non-episode") to justify the use of this duration in the definition of pain free episode.

**Objectives:**

Two clinical populations were followed weekly over one year making it possible 1) to determine the maximum numbers in a row of weeks without LBP, 2) to determine the prevalence of non-episodes throughout a one-year period, and 3) to find the prevalence of patients who reported to be in a non-episode of LBP at the end of the study.

**Methods:**

Secondary data were used from two recent clinical studies, in which weekly automated text messages (SMSes) had been collected on the number of days with LBP in the preceding week for one year. Weeks with 0 days of LBP were defined as "zero-weeks" and four zero-weeks in a row were defined as a period without LBP (a"non-episode") according to de Vet et al's suggestion. The study participants, all from the secondary care sector, consisted of: study 1) patients with LBP and Magnetic Resonance Imaging-identified Modic changes and study 2) patients without obvious acute disc problems, Modic changes or other pathologies, who therefore were assumed to have non-specific LBP. Both studies were two-armed intervention studies without a significant difference in outcome between intervention groups. The number of zero-weeks was identified in each participant. Thereafter the numbers of participants who reported at least one non-episode during the study period were identified. Finally, the numbers of participants who had a non-episode at the end of the study were counted. Estimates are reported with their 95% confidence intervals.

**Results:**

The numbers of participants included in the analyses were 80 and 209. Most commonly, no zero weeks were reported, by 65% (55-75) and 56% (49-63) of patients, respectively. The percentages of study participants with at least one non-episode at some time during the course of the study were 20% (11-29) and 18% (15-21. The percentages of participants who were identified as being in a non-episode at the time of the last week of the study were, 5% (95% CI: 0-10) and 4% (1-7) respectively.

**Conclusions:**

The vast majority of these secondary care sector patients had a profile of more or less constant LBP. The estimates for non-episodes during the study period and at the end of the study were very similar for participants with LBP who also had Modic changes and those with non-specific LBP. It is possible that a definition of pain-free periods is pointless in patients seeking care in the secondary care sector.

## Background

The study of low back pain (LBP) would benefit from a clear definition of when LBP is absent and when it is present. As discussed by de Vet et al. [1], this is necessary when investigating the development of LBP in relation to risk factors, and when defining inclusion criteria for patients wanted for participation in clinical studies. In the first instance, it would be necessary to include people who are disease-free whereas in the second case, it must be clear who has and who does not have LBP. In the long-term follow-up of clinical studies it may also be necessary to establish whether patients have recovered and whether new episodes of LBP have occurred and if so, whether these were persistent or time-limited episodes only.

The identification of the end of an episode of LBP or of the onset of a new episode of LBP obviously depends on the presence of a period of absence of LBP (a "non-episode") and a clear demarcation between the period of pain and the period without pain. Previously this concept of episodes was not considered important, as LBP was perceived as a short-lasting and benign condition for most (acute LBP), with some people experiencing the pain somewhat longer (subacute LBP), and only an unfortunate minority persisting into chronicity [2], rather like a benign infectious disease such as the common cold. This concept invites the idea that LBP is abnormal and absence of LBP normal.

However, over the past years it has become increasingly acknowledged that LBP is a recurring condition, an episodic disease [3], rather to be likened to asthma; not always apparent but always ready to appear. Other more dynamic definitions and descriptions of the course of LBP have therefore emerged, taking into account the varied patterns over time [4-6].

This approach is an improvement, but as de Vet et al. point out [1], an evidence-based definition of "the episode" is missing. They propose that episodes should be divided into "care-based" and "pain-based". In relation to the latter, in a literature review they found a number of definitions of the pain-free period ranging from 1 month to 1 year that had been used in studies, whereas in other studies this pain-free period had not even been specified. They tentatively proposed that a definition of an episode of LBP would be preceded and followed by 1 month without LBP whilst pointing out that this definition would be "most applicable in patients who do indeed have clear periods of LBP, alternating with LBP-free periods". This definition of "recovery" has been recommended as an interim definition in a systematic review of the literature [7].

It would be an advantage if researchers could agree on suitable definitions for episodes and non-episodes. However, such definitions should be evidence-based, which none of the definitions previously used in the literature, as identified by de Vet et al., were found to be. As we had access to prospective data on LBP collected weekly over a period of one year from two randomized clinical trials, we decided to investigate the feasibility of de Vet et al's proposed definition of the period of non-LBP, i.e. absence of pain for at least one month.

The overall aim of this study was to study the pattern of "non-episodes" in two clinical study samples over a one-year period. Specifically:

1) To determine the maximum number of consecutive weeks without LBP ("zero-weeks"),

2) To calculate the percentage of participants who, at least once, reported to have had no episode of LBP lasting for at least 4 consecutive weeks (i.e. a non-episode), and

3) To calculate the percentage of participants who reported to have a non-episode at the end of the study.

## Method

Data were obtained from two clinical studies carried out in Denmark in the secondary care sector. The studies and inclusion criteria are briefly described below and the main results will be reported elsewhere. This report deals with secondary analyses of some of the data obtained in these studies.

### Studies

The studies were randomized clinical trials that took place at a specialized outpatient spine clinic in a public hospital in Denmark during 2007-09, testing the effect of different treatments for LBP. Patients considered for these studies had been referred from local general practitioners and chiropractors. All patients who entered the clinic, providing that they fulfilled some minimal criteria, were screened through an initial questionnaire and had a magnetic resonance imaging (MRI) taken. The criteria were: LBP or leg pain of ≥ 3 out of 11 on numerical rating scale, duration of 2-12 months and age between 18-60 years. Both studies formed the basis for Ph.D. projects, none of which has as yet been published.

Included in study 1 (ClinicalTrials.gov identification number NCT00454792) were those who had localized LBP with MRI-defined vertebral endplate changes (Modic changes). There were two treatment arms, one operating according to the "don't worry-keep active" concept (an exercise program for 10 weeks), whereas the other was based on the assumption that Modic changes consist of inflamed micro-fractures of the vertebral body and that some rest and absence of hard work would be necessary for healing. There was no discernable difference between study groups at baseline (tested variables included age, gender, BMI, smoking, type of occupation, education, sick leave, LBP intensity, activity limitation, general health and depression) and preliminary analyses did not reveal any difference in outcome in relation to LBP intensity, activity limitation, general health and number of days with troublesome pain. There was also no difference in number of drop-outs after one year (data not shown but to be reported elsewhere), making it possible to analyze the data for both intervention groups together.

The second trial (ClinicalTrials.gov identification number NCT00459433) included participants who were not eligible for study 1 and who, in addition, did not have an obvious acute disc problem nor any other obvious pathology, and therefore were assumed to be suffering from non-specific LBP. Treatment in the second study was based on the bio-psycho-social model with the intervention group receiving a needs-based psychosocial treatment in addition to the treatment for the control group that consisted of the clinic's "usual" approach consisting of a thorough examination, explanations, training/spinal manipulation/medication as needed, and advice on continued management. No differences were noted between the treatment and control groups at baseline (tested variables included age, gender, educational level, dispute about work accidents, previous low back pain, psychosocial profile, LBP intensity and activity limitation) and there was also no difference in outcome in relation to number of days with troublesome pain LBP intensity and number of days with reduced activity of daily living. There were also no difference in amount of missing data between these at follow-up (data not shown but to be reported elsewhere), which made it possible to analyze the data for both treatment arms together in this study as well.

For ease of reporting these studies will be referred to as "Study 1" (study on patients with Modic changes) and "Study 2" (study on patients with non-specific LBP).

### Weekly data collection with text messages

All participants in these studies filled out questionnaires at baseline and received follow-up questionnaires but, for the purpose of the present report, only the collection of weekly text message data on LBP (described below) will be used in the analyses.

All participants had been asked at the beginning of the study if they had a mobile phone, if they could use its text message function, and if they would be willing to receive questions relating to their LBP status and to send answers in the form of text messages relating to the study over a period of twelve months. Participants who were suitable and accepted participation were included in the respective studies. Ethics and data management approvals (information available from the authors on request) together with trial registrations were obtained for both studies. An instruction was given to the participants on the procedure and on how to answer the questions. Automated text messages, using the SMS-Track-Questionnaire [8], were thereafter sent to them every week, containing standardized questions on LBP, with definitions that referred to LBP being bothersome (in Danish: "causing problems"). The concept of bothersomeness has been studied and found to correlate well with pain intensity, function and quality of life [9-11]. In a previous Danish study, the degree of bothersomeness was highly correlated with pain intensity [12]. With this question it was hoped that the occasional twinge or discomfort would not be included but that mainly LBP that was felt to have a consequence upon daily living would be reported.

The question on LBP was: "With a number between 0-7, please answer how many days in the past week you have had problems with your low back?" The answers were given in a reply message requiring only a number, referring to the number of days with LBP, e.g. "0" if there had been no days with problems during the preceding week or "3" if the week had contained three days with LBP problems.

### User friendliness and validity of text-messaging data

According to an inter-method reliability study performed within Study 2, participants had not felt the repetitive SMS-Track questions inconvenient. Upon asking the participants about the acceptable number of SMS questions, all had agreed that three weekly questions would have been acceptable as opposed to the two questions included in that study [13]. The same study illustrated also the effect of memory decay when collecting retrospective data on pain, indicating the need to ask questions at short intervals rather than at long intervals. A Swedish study found the SMS-Track system user friendly, yielding high response rates unaffected by season and that the drop-outs were not the young men that are often seen in studies with some follow up time [14].

### Data management and analysis of data

**Figure 1:**
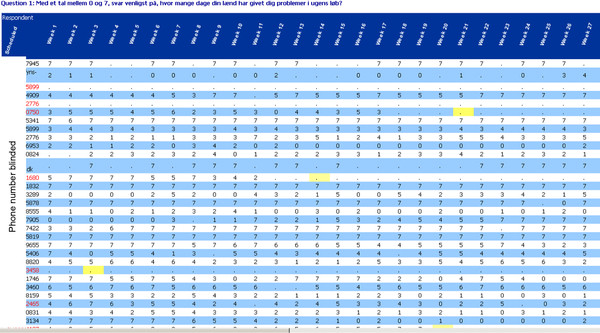
**Example of a data file with SMS responses**. Each participant's (y-axis) weekly responses (x-axis) have been automatically entered and are assessable to the researcher in real time. The data can be transferred to other software programs for statistical analysis and the file can also be merged with other data files.

Each week a text message was sent with the SMS-Track-Questionnaire system. The participant's response message went automatically into a computer file to be used for data analysis. This file resembles a spread-sheet, with each participant's weekly answers listed in a horizontal row, as shown in Figure 1. The Danish Data Protection Agency considered the built-in encryption of the SMS systems in all telecommunication companies in Denmark as sufficient protection of participants' data when data were exchanged between participants and the server. Once the data had arrived to the server used for this purpose, they were also password protected, again in such a way as to secure approval. Technical support was provided by the company providing this system [8].

In the present study, print-outs were made of these spread-sheets to allow for manual analysis. Participants in the present study, who failed to respond at least 50% of the time, were excluded from the analysis. For each individual, the number of days with LBP per week was identified, with 0 days of LBP being defined as a zero-week. All other numbers (1-7) were considered to represent weeks with at least some LBP. Missing cells were considered to represent a week with LBP in order not to overestimate the presence of zero-weeks. Visual inspection revealed that missing cells predominantly were surrounded by reports of weeks with LBP rather than zero-weeks, indicating that missing data were more likely to represent presence of LBP than absence of LBP.

To address the three study objectives, analysis was carried out in the following manner. First, we identified the spread of data for the maximum number of zero-weeks per individual. Thereafter, the number of individuals was identified, who at some time during the study had reported at least 4 weeks in a row without LBP. A minimum of 4 zero-weeks in a row anywhere during the study period was considered a non-episode, as defined by de Vet et al. [1]. Finally, those individuals were identified who could be defined as having a non-episode at the end of the study, i.e. counting those who reported at least 4 zero-weeks in a row, counting backwards from the last week of the study. If no information was available for the last week, the second last week was used as a starting point instead.

A post hoc/secondary analysis was undertaken, in which a more lenient definition of missing cells was used to identify study participants with at least one non-episode or who were LBP free for at least four weeks at the end of the study. When a missing cell was found within 4 weeks from a "real" zero-week, the missing cell was considered to be a zero-week.

Results have been reported as percentages with 95% confidence intervals throughout the text. Non-overlapping intervals were considered to indicate statistically significant differences between estimates.

## Results

### Description of study samples

The numbers of participants who entered the studies were 100 and 241. Twenty participants did not participate in the text-message data collection, because they dropped out at the start in Study 1, and 32 participants in Study 2 did not provide sufficient weekly data for at least 50% of the weeks, and were therefore excluded from our analyses. Therefore, study 1 included data on 80 individuals and study 2 on 209. The data will be reported in this order. A further description of the study samples is provided in Table 1.

**Table 1:** Baseline description of the two clinical study samples of patients with low back pain (LBP)

Population	Study 1	Study 2
	**LBP with Modic changes**	**Non-specific LBP**

**Number of participants**	100	241

**Age mean**	46	38

**Age range**	21-61	18-60

**Proportion women**	68%	54%

**Mean LBP according to Low Back Pain Rating Scale (0-30)**	18	18

**Mean LBP according to Numeric Rating Scale (0-10)**	5.3	4.9

**Mean leg pain according to Low Back Pain Rating Scale (0-30)**	12	10

**Mean disability according to Low Back Pain Rating Scale (0-100)**	47	50

**Duration of LBP at baseline** **(inclusion criterion)**	3-12 months	3-12 months

***Type of occupation (mainly):***		

**Sitting**	17%	12%

**Mostly walking**	40%	19%

**Walking and some lifting**	23%	28%

**Heavy work**	20%	40%

It was not common that data were missing often or for longer periods. Among the participants in Study 1, 72% reported every week and in study 2, this was done by 40%. For detailed information see Table 2. The higher compliance in study 1 was due to a firmer control of non-responders who were contacted with a re-explanation of the procedure early in the course.

**Table 2:** Description of missing information in two populations followed over 1 year with weekly text messages

	Study 1	Study 2
**Number of weeks with no text-message response**	**Number of participants (%)**	**Number of participants (%)**

0	58 (72.5)	83 (40)

1-5	17 (21)	84 (40)

6-10	3 (4)	15 (7)

11-26	2 (2.5)	27 (13)

27 + *		

Study 1 (N = 80) consisted of participants with Modic changes and study 2 (N = 209) included participants with non-specific low back pain.

* Participants with 27 or more missing weeks had been excluded from the analyses already from the beginning

### Participants and non-participants

Various comparisons between drop-outs and participants, and between highly and less compliant participants showed no obvious differences on the baseline data (data not shown but to be reported elsewhere).

### The percentages of participants who reported to have had weeks without LBP (zero-weeks)

Over the study period, 65% (55-75) and 56% (49-63), respectively, reported no zero-weeks at all. For those reporting 1 to 3 zero weeks in a row, see Table 3.

**Table 3:** Percentages of participants with at least 4 zero-weeks in a row (non-episodes)

	Study 1 LBP with Modic changes	Study 2 Non-specific LBP
**Maximum number of zero-weeks in a row per person**	**% (95% CI)** **N = 80**	**% (95% CI)** **N = 209**

**0**	65 (54-76)	56 (49-63)

**1**	11 (4-18)	13 (8-18)

**2**	4 (4-4)	6 (6-6)

**3**	0 -	6 (6-6)

**4**	1 (1-1)	3 (3-3)

**5**	5 (5-5)	1 (1-1)

**6**	2 (2-2)	1 (1-1)

**7**	1 (1-1)	1 (1-1)

**8**	4 (4-4)	0 -

**9 or more**	6 (6-6)	11 (7-15)

### The percentages of participants who at some time during the follow-up period reported to have at least four weeks in a row without LBP (non-episodes)

The percentages of participants with at least one non-episode during the course of the study were 20% (11-29) and 18% (13-23), respectively. For more detailed information on the duration of these non-episodes, see Table 3. The more lenient post hoc analysis added only 1 case to study 1 and 6 cases to study 2 and was therefore not further taken into consideration.

### The percentages of participants who reported to have at least four weeks in a row without LBP (non-episodes) at the end of the study

At the end of the 12 months follow up, the percentages of participants who could be defined as having a non-episode were 5% (0-10) and 4% (1-7), respectively. The more lenient post hoc analysis added no cases to these estimates.

## Discussion

### Absence of LBP during the entire study period and at the end of the study

The presence of zero-weeks was relatively rare over the entire study period. More than half of the study participants reported none at all. As a consequence, non-episodes were rare. Only approximately 20% of the participants reported at least one non-episode sometime during a period of one year, and at the time for the 1-year follow-up only about 5% of the participants experienced 4 weeks in a row without LBP. Hence, if the presence of a non-episode would be the criterion for recovery, the outcome looks quite gloomy for these participants. Interestingly, however, there is also no clear evidence-based definition of recovery in the literature [14].

Obviously, similar studies need to be undertaken in other clinical populations, in order to establish if there is, at a given time, a sufficiently large number of people having four-week long non-episodes of LBP, justifying its use as a definition of recovery and as a demarcation for episodes of LBP.

It was also noted that most weeks during the one-year period were weeks with at least some days of LBP. Therefore, the results of this study suggest that LBP may be a persistent condition rather than a recurring one, at least over a period of 1 year and in the secondary care sector. Another possibility is that it *is *a recurring condition but with shorter ups and downs than the definitions used in this study.

Our findings do not necessarily detract from the usefulness of the proposed definition of a non-episode of LBP [1]. However, to determine its validity, it would be necessary to study the prevalence of non-episodes of LBP in various populations and to do so with weekly data collection.

As it is possible that the non-episode profiles would differ between populations, such studies would have to be performed in different well-defined populations. As it is also likely that different methods of data collection (such as retrospective questionnaires, diaries, frequent telephone interviews, or as in this study, frequent text-messaging) would result in different profiles, such studies should use the same method of data collection, to make comparisons between studies and study populations credible.

If the frequency of non-episodes is found to vary markedly in different populations, it may well become a useful definition to delineate differences - or similarities - between different types of patients with LBP. Our results showed clearly that the two study groups from the secondary care sector, patients with LBP accompanied by Modic changes and those with non-specific LBP, resemble each other greatly, in that absence of LBP is equally uncommon.

However, if 4-weekly non-episodes are equally rare in other populations, then "new" events of LBP may not be a suitable inclusion criterion in clinical studies. In fact, definitions of a non-episode and a new episode of LBP may not even be relevant. In such a case, some other more objective and clinically relevant inclusion criterion may be more useful, such as a new episode of seeking care.

### Strengths and limitations of the study

The findings of these two studies appear credible in that the results are similar.

In studies there will always be some differences between the participants/non-participants and between compliers/non-compliers on known or unknown variables but it is difficult to judge how, if at all, such differences would influence the results. However, no obvious differences were found in these two studies.

The main advantage with this study is that data were collected weekly with a simple method, using mobile phones and text-messaging. This meant that the recall period was so short that memory decay would be unlikely. Frequent reporting also avoids the problem of back-filling information, such as has been noted with diaries, when used for longer periods [15]. More detailed information could have been sought in this study, which could have improved our knowledge of the LBP pattern. However, too frequent or lengthy questions could be tiresome for the participants, resulting in a poorer response rate. Therefore, text-message questions should be few, well selected and easy to answer.

We had opted for a brief definition of LBP with some consequence, i.e. that it should be considered to be a "problem" in order to count. Participants generally found no problems in answering these questions and a follow-up interview in one of the populations (study 2) confirmed that they had been well accepted [13]. The high participation rate, for those who "passed" the > 50% compliance criterion, also indicates user friendliness.

In our study the severity or extent of the pain was not recorded. Other, more detailed definitions, with various specific threshold levels for LBP could produce different results. Nevertheless, we consider our definition of a non-episode to be realistic. Surely, patients who report that they have an entire pain-free week feel that they have... no LBP. It is of course the patient who should judge his/her state and not the researcher, based on some intuitive threshold value obtained from a pain or disability scale.

## Conclusions

The definition of a non-episode (four consecutive weeks without LBP) previously suggested by a team of researchers [1] was tested in terms of prevalence of findings in two clinical populations with the following results:

• It was more common than not for participants in these two studies to report at least some LBP during the majority of weeks over one year.

• Not surprisingly, it was therefore uncommon that they were free from LBP at least four weeks in a row at some time during the study period. It was even less common to have been pain-free for at least four weeks in a row at the end of the 1-yr study.

• These findings demonstrate the need to develop evidence-based criteria for non-episodes of LBP in different populations.

• In addition, these results indicate that, at least for patients in the secondary care sector, both specific LBP (LBP with Modic changes) and non-specific LBP (patients explicitly screened for absence of pathological findings) should be described as constant rather than episodic conditions.

## Competing interests

The authors declare that they have no competing interests.

## Authors' contributions

CLY instigated this work and performed the analysis. RKJ was the project administrator for Study 1 described in this article. All the authors were involved in writing the manuscript, and have approved the final version.

## References

[B1] de Vet HCW Heymans MW Dunn KM Pope DP van der Beek AJ Macfarlane GJ Bouter LM Croft PR Episodes of low back pain. A proposal for uniform definitions to be used in research Spine 2002 27 2409 2416 10.1097/00007632-200211010-00016 12438991

[B2] Spitzer WO LeBlanc FE Dupuis M Scientific approach to the assessment and management of activity-related spinal disorder. A monograph for clinicians Spine 1987 12 S16 S21 2961086

[B3] Stanton TR Latimer J Maher CG Hancock MJ How do we define the condition 'recurrent low back pain'? A systematic review Eur Spine J 2010 19 533 539 10.1007/s00586-009-1214-3 19921522 PMC2899839

[B4] v Korff M Dunn KM Chronic pain reconsidered Pain 2008 138 267 276 10.1016/j.pain.2007.12.010 18226858 PMC2613775

[B5] Tamcan O Mannion AF Eisenring C Horisberger B Elfering A Muller U The course of chronic and recurrent low back pain in the general population Pain 2010 150 3 451 457 10.1016/j.pain.2010.05.019 20591572

[B6] Kongsted A Leboeuf-Yde C The Nordic back pan subpopulation program - individual patterns of low back pain established by means of text messaging: a longitudinal pilot study Chiropr Osteopat 2009 17 11 10.1186/1746-1340-17-11 19919715 PMC2781014

[B7] Stanton TS Latimer J Maher CG Hancock MJ A modified Delphi approach to standardize low back pain recurrence terminology Eur Spine J 2011 20 744 752 10.1007/s00586-010-1671-8 21193932 PMC3082681

[B8] SMS-Track http://www.sms-track.com

[B9] Daltroy LH Cats-Baril WL Katz JN Fossel AH Liang MH The North American spine society lumbar spine outcome assessment Instrument: reliability and validity tests Spine (Phila Pa 1976) 1996 21 6 741 749 10.1097/00007632-199603150-00017 8882698

[B10] Deyo RA Battie M Beurskens AJ Bombardier C Croft P Koes B Malmivaara A Roland M Von Korff M Waddell G Outcome measures for low back pain research. A proposal for standardized use Spine 1998 23 18 2003 2013 10.1097/00007632-199809150-00018 9779535

[B11] Patrick DL Deyo RA Atlas SJ Singer DE Chapin A Keller RB Assessing health-related quality of life in patients with sciatica Spine (Phila Pa 1976) 1995 20 17 1899 1908 discussion 1909 10.1097/00007632-199509000-00011 8560339

[B12] Kongsted A Leboeuf-Yde C The Nordic back pain subpopulation program: course patterns established through weekly follow-ups in patients treated for low back pain Chiropr Osteopathy 2010 18 2 10.1186/1746-1340-18-2 PMC2820484 20150994

[B13] Johansen B Wedderkopp N Comparison between data obtained through real-time data capture by SMS and a retrospective telephone interview Chiropr Osteop 2010 18 10 10.1186/1746-1340-18-10 PMC2883994 20500900

[B14] Kamper SJ Stanton TR Williams CM Maher CG Hush JM How is recovery from low back pain measured? A systematic review of the literature Eur Spine Jdoi:10.1007/s00586-010-1477-8 10.1007/s00586-010-1477-8 PMC3036032 20552378

[B15] Stone AA Shiffman Schwartz S Broderick JE Hufford MR Patient compliance with paper and electronic diaries Control Clin Trials 2003 24 2 182 199 10.1016/S0197-2456(02)00320-3 12689739

